# Retrospective analysis of the role of retrobulbar amphotericin-B injection in the management of COVID-19 associated rhino-orbito-cerebral-mucormycosis

**DOI:** 10.11604/pamj.2022.42.312.34757

**Published:** 2022-08-25

**Authors:** Rekha Yadav, Siddharth Madan, Jolly Rohatgi, Anam Ansari, Rahul Sharma, Priyanka Gautam, Puja Rai, Gopal Krushna Das, Pramod Kumar Sahu, Vipin Arora

**Affiliations:** 1 Department of Ophthalmology, University College of Medical Sciences and Associated Guru Teg Bahadur Hospital, Delhi, India,; 2 Department of Community Medicine, University College of Medical Sciences and Associated Guru Teg Bahadur Hospital, University of Delhi, Delhi, India,; 3 Department of Otorhinolaryngology, University College of Medical Sciences and Associated Guru Teg Bahadur Hospital, University of Delhi, Delhi, India

**Keywords:** COVID-19, retrobulbar amphotericin B, orbital exenteration

## Abstract

**Introduction:**

Rhino-orbito-cerebral-mucormycosis (ROCM) is the most common form of mucormycosis observed during the second wave of COVID-19 where a steep rise in the number of cases was seen. The orbital form is almost always associated with fungal sinusitis. Among the various treatment modalities available, the role of retrobulbar Amphotericin-B injections is under-reported. This study is conducted to determine the role of transcutaneous retrobulbar amphotericin-B (TRAMB) in the management of COVID-19 associated ROCM.

**Methods:**

a retrospective analysis of 61 patients of COVID-19 associated ROCM was done, who met the inclusion criteria and presented to a tertiary care center, between May to August 2021. These patients were administered TRAMB (deoxycholate/emulsion form) along-with systemic amphotericin B. All the patients were evaluated for clinical improvement.

**Results:**

out of 61 patients, 58 (95.08%) showed overall improvement. 40 patients (65.57%) stabilized or improved clinically and 3 patients succumbed to the illness due to advanced systemic mucormycosis and acute kidney failure. Sixteen out of 58 patients underwent orbital exenteration. Out of remaining 43 patients, 35 showed complete recovery of orbital and ocular disease and the disease stabilized in eight patients. Seven patients demonstrated TRAMB associated ocular complications which however completely resolved in six patients.

**Conclusion:**

to the best of the author´s knowledge, regression of orbital mucormycosis with improvement in ptosis, proptosis, ocular motility and stabilization of visual acuity are scarcely reported in literature. Further TRAMB as a globe non-deforming treatment modality is an option available for ROCM.

## Introduction

Invasive rhino-orbito-cerebral mucormycosis (ROCM) is a potentially fatal disease caused by fungi of the genus Rhizopus, Mucor, Rhizomucor, Cunninghamella and Absidia which belong to order-Mucorales; Class-Zygomycetes [1]. *Rhizopus oryzae* is the commonest type which accounts for nearly 60% cases of mucormycosis in humans and about 90% of cases that develop ROCM [2, 3]. Mortality is high if the treatment is not initiated timely and promptly. Several underlying factors predispose an individual to develop invasive fungal sinusitis. These include immunosuppressed state, diabetes mellitus, new onset hyperglycemia, steroid-induced hyperglycemia, hematologic malignancy, or transplant recipients [4-9]. Other risk factors include hypoxia, metabolic acidosis, diabetic ketoacidosis (DKA), increase in serum iron levels/ferritin, immunosuppression due to a reduction in the phagocytic activity of leucocytes following severe acute respiratory syndrome coronavirus-2 (SARS-CoV-2) infection or steroid administration apart from an increased duration of hospital stay [4-8]. With the advent of COVID-19 and indiscriminate use of steroids as a part of treatment regimen for COVID-19, a tremendous increase in mucormycosis cases was observed in India.

COVID-19 is primarily a viral respiratory and vascular illness that targets the respiratory and vascular systems. The spike protein of the SARS-CoV-2 binds to the the angiotensin converting enzyme 2 (ACE2) receptors in the lungs and other tissues of the body. This binding exposes the fusion sequences resulting in the fusion of the cell membranes and novel coronavirus. The virus enters the host cell subsequently and begins to replicate [10]. The clinical manifestations of the COVID-19 vary from an asymptomatic disease to life threatening acute respiratory failure and/or multiple organ failure [11]. A cytokine-induced inflammatory storm observed is indicated by an elevation in the neutrophil-lymphocyte and platelet-to-lymphocyte ratio [12]. The incidence of mucormycosis is on a rise and the number of cases reached epidemic proportions amidst COVID-19. About 0.14 per 1000 cases in India got reported in the year 2019-2020 when compared with developed countries where it varies from 0.005 to 1.7 per million population [13-16]. A worldwide surge in COVID-19 associated mucormycosis especially in India is seen due to hypoxia, new onset or steroid induced hyperglycemia, metabolic and diabetic ketoacidosis, increase in the levels of serum ferritin and accompanying leucopenia with a reduction in the phagocytic activity of the white blood cells. COVID-19 associated mucormycosis is mostly observed in patients who are immunocompromised and have raised blood sugar levels, uncontrolled diabetes mellitus, underlying malignancies, transplant recipients and those with prolonged neutropenia [17].

Unlike COVID-19, individuals infected with human immunodeficiency virus (HIV) who develop mucormycosis had underlying risk factors that comprised of parenteral drug use, corticosteroid use, CD4+ counts <50 cells/mm^3^ and transient neutropenia [18]. HIV/AIDS per se does not seem to be a significant risk factor as is evident from the paucity of cases of mucormycosis observed in individuals suffering from HIV/AIDS. In vitro studies have shown that neutrophils are required for inhibiting the proliferation of fungal spores. Further, the frequency of mucormycosis in HIV infected patients varies from 0.12-4.9% in clinical and autopsy series [18]. The co-infection of mucormycosis with HIV/AIDS was observed rarely when the only predisposing factor was infection with HIV. Low level of CD4+ counts with the presence of additional risk factors like intravenous drug use and transient neutropenia should raise the suspicion of the development of mucormycosis in an individual [19-22]. Seventeen patients having liver cirrhosis due to Hepatitis C virus (HCV), hepatitis B virus (HBV), autoimmune and alcoholic diseases developed mucormycosis as per a study [23, 24]. Eight cases had DM as a co-morbidity while 3 patients were on steroids as risk factors. It is also proposed that patients with liver cirrhosis might develop hypersplenism that results in neutropenia and predisposes to the development of mucormycosis. In the presence of normal total leucocyte count, a reduction in the neutrophil activity might be an underlying pathogenetic mechanism [25]. Invasive mucormycosis has a strong association with DM and very rarely with liver cirrhosis.

Rhino-orbito-cerebral mucormycosis being the commonest form has various subtypes including rhino-nasal, rhino-orbital or rhino-orbito-cerebral based on the tissue that is involved. Rhino-orbital infection begins when fungal spores that are inhaled invade the nasal mucosa, and sinusitis develops [9]. Orbital involvement occurs as a result of invasion of the orbital wall from the paranasal sinuses [7]. The management approach is directed towards early sinonasal debridement apart from systemic antifungal therapy [26]. Amphotericin B (AMB) is the gold standard for the systemic treatment of mucormycosis. The diffusion of AMB is slow into tissues due to its high protein-binding capacity and a larger molecular weight [27-31]. Moreover, due to the angio-invasive nature of the disease, reduced vascular supply to the affected tissues limits the penetration of the systemic antifungal drugs [27-31]. For this reason, local administration of infected areas with AMB is used in conjunction with surgery and systemic therapy. Transcutaneous Retrobulbar Amphotericin B (TRAMB) injection has been reported as a minimally invasive, globe-sparing intervention for orbital aspergillosis [29-32]. Progressive orbital involvement in eyes without any possibility of visual recovery and an attempt to prevent intracranial extension despite surgical debridement necessitates orbital exenteration [33]. There are limited number of studies in literature that evaluated the response of retrobulbar injection of AMB in ROCM. No definite protocol is postulated for the treatment of orbital presentation of ROCM. The purpose of this study is to evaluate the outcome of RAMB injection in patients with ROCM and the role of orbital exenteration in non-responsive cases.

**Objectives:** the primary objective of this study is to evaluate the role of retrobulbar Amphotericin B injection in patients with Rhino-orbito-cerebral mucormycosis. Further the study also assessed the need for orbital exenteration for orbital involvement in ROCM in patients who underwent preceding or simultaneous sinonasal debridement in COVID-19 associated ROCM.

## Methods

**Study design:** this was a retrospective analysis of cases who presented to the hospital between May 2021 to August 2021.

**Setting:** the study was conducted in the Department of ophthalmology and Department of otorhinolaryngology (ENT) at a tertiary care hospital in Delhi, India.

**Participants:** the patients with COVID-19 associated ROCM during COVID-19 pandemic with or without a definitive history of COVID-19 who received RAMB injections were included in the study.

**Study size:** all patients (n=60) with ROCM with a visual acuity of no perception of light to finger counting at at-least 6 meters, with or without ptosis, proptosis, limitation of ocular motility to total ophthalmoplegia with radiological features suggestive of invasive fungal sinusitis with orbital involvement were included. All the subjects who were included gave a written and informed consent for administration of RAMB injection with or without sinonasal debridement and orbital exenteration. Patients who did not give consent for retrobulbar injection of AMB or for orbital exenteration were excluded.

**Variables:** the cases included in the study were confirmed/suspected/recovered cases of COVID-19 associated ROCM showing clinical and radiological evidence of orbital involvement. All patients presenting to the casualty of Guru Teg Bahadur Hospital, Delhi, India with clinical features suspicious of mucormycosis were examined. The history of COVID-19 was elucidated and the patients were labelled as recovered/suspected/active cases of COVID-19 based on the reports of the investigations and reverse transcriptase polymerase chain reaction (RTPCR)/rapid antigen test (RAT) report available with them at that point of time. The suspected cases without any report and those with a definitive report of being positive for SARS-CoV-2 were admitted in COVID-19 suspect ward of the hospital and RTPCR testing was done in all cases. A detailed ocular and Ear, Nose and Throat (ENT) examination and examination by the medicine department was done on admission. Diagnostic nasal endoscopy was performed in all cases, the examination findings documented and a sample from the sinus/nasal cavity was sent for KOH testing. Contrast enhanced (CE)/non-contrast computed tomography scan (CE/NCCT scan) of the Brain, orbit and paranasal sinuses was done in all patients based on their kidney function test (KFT) report. If a patient was already carrying their recent magnetic resonance imaging (MRI)/CT reports, the same was taken as a baseline for further management. The necessary history during the admission for COVID-19 prior to the development of mucormycosis, with the risk factors in the form of oxygen supplementation, steroid intake, diabetes and other co-morbidities were tabulated. Comprehensive ophthalmic evaluation included assessment of the visual acuity, pupil reaction, anterior and posterior segment evaluation including dilated fundus evaluation, ocular motility assessment, measurement of proptosis, ptosis and its severity, and the observations were tabulated (Table 1, Table 2). The staging of ROCM was based on the staging proposed by Honavar *et al*. [34]. Stage 3 comprised of patients having orbital involvement. Stage 3a included subjects with involvement of the nasolacrimal duct, medial orbit however the vision remained unaffected. Individuals with stage 3b had diffuse orbital involvement (> 1 quadrant or > 2 structures) with vision unaffected. Stage 3c had individuals with central retinal artery or ophthalmic artery occlusion, superior ophthalmic vein thrombosis; involvement of superior orbital fissure, inferior orbital fissure, orbital apex, loss of vision). Patients with stage 3d had bilateral orbital involvement. Individuals with stage 4 had involvement of the central nervous system.

**Table 1:** demographic profile of the patients

Parameter	No. of patients in various stages			
	3a	3b	3c	4
Total Number of patients	10	9	21	21
Proptosis	2	2	17	17
Ptosis	3	4	19	18
EOM (0 to -4)	1	1	2	3
Total ophthalmoplegia	3	3	17	15
**VISUAL ACUITY- right eye (PRESENTATION)**				
PL negative	0	0	5	7
PL +ve HMCF	0	0	1	1
1/60-3/60	2	1	5	3
6/60-6/36	6	8	9	7
6/24-6/9	2	0	1	3
6/6	0	0	0	0
**VISUAL ACUITY- left eye (PRESENTATION)**				
PL negative	3	1	10	7
PL +ve HMCF	1	1	0	2
1/60-3/60	1	2	4	3
6/60-6/36	5	5	7	8
6/24-6/9	0	0	0	1
6/6	0	0	0	0
**VISUAL ACUITY- right eye (LAST)**				
PL negative	0	0	5	7
PL +ve HMCF	0	0	1	1
1/60-3/60	2	0	5	3
6/60-6/36	6	9	9	7
6/24-6/9	2	0	1	3
6/6	0	0	0	0
**VISUAL ACUITY- left eye (LAST)**				
PL negative	3	1	10	7
PL +ve HMCF	1	1	0	2
1/60-3/60	1	2	4	3
6/60-6/36	5	5	7	8
6/24-6/9	0	0	0	1
6/6	0	0	0	0
**FUNDUS- right eye**				
NAD	10	9	14	13
Media Hazy	0	0	4	3
Optic Atrophy/ CRAO/ Disc Edema	0	0	3	5
**FUNDUS- left eye**				
NAD	3	0	5	0
Media Hazy	7	9	14	13
Optic Atrophy/ CRAO/ Disc Edema	0	0	2	8

**Table 2:** risk factors, otorhinolaryngology (ENT) and imaging findings

**Age (average age) in years**	25-77 (51.4)
**Gender**	
	Male- 39
	Female- 22
**Risk factors**	
Diabetes	49/61 (80.32%)
Oral cavity	11/61 (18.03%)
Nasal cavity	55/61 (90.16%)
Positive for SARS-CoV-2	32/61 (52.45%)
**Diagnostic Nasal Endoscopy**	
Congestion	9/61 (14.75%)
Secretion	41/61 (67.21%)
Crusting	33/61 (54.09%)
Black crusting	12/61 (19.67%)
**CT scan/ MRI**	**Number of patients showing involvement of various paranasal sinuses**
Ethmoidal-Unilateral/ bilateral	14/61 (22.95%) and 15/61 (24.59%)
Sphenoidal- unilateral/bilateral	4/61 (6.55%) and 11/61 (18.03%)
Maxillary- unilateral/ bilateral	16/61 (26.22%) and 12/61 (19.67%)
Frontal- unilateral/ bilateral	9/61 (14.75%) and 10/61 (16.39%)
Pansinusitis	17/61 (27.86%) and 9/61 (14.75%)
**Orbital involvement**	
Inferior	20/61 (32.78%)
Medial	20/61 (32.78%)
Floor	5/61 (8.19%)
**Others**	
Pterygopalatine fossa/ IT fossa	11/61 (18.03%)
Intraconal involvement	9/61 (14.75%)
Extraconal involvement	36/61 (59.01%)
Intracranial extensions	24/61 (39.34%)
Apex involvement	32/61 (52.45%)

**Figure 1:**
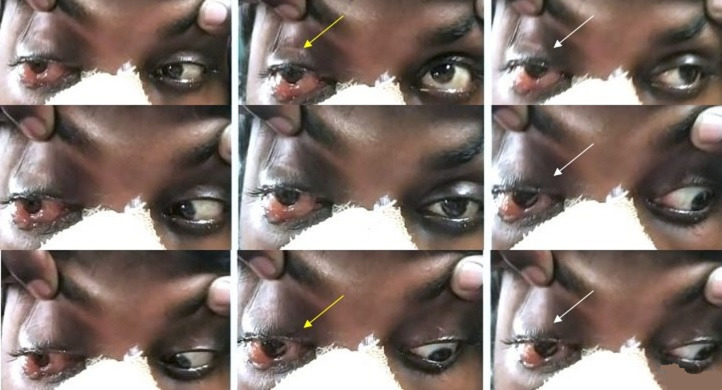
a patient presented with proptosis, chemosis, ophthalmoplegia (yellow arrow showing limitation in upgaze and downgaze in the right eye and white arrow showing limitation in levoelevation, levoversion and levodepression in the right eye)

**Figure 2:**
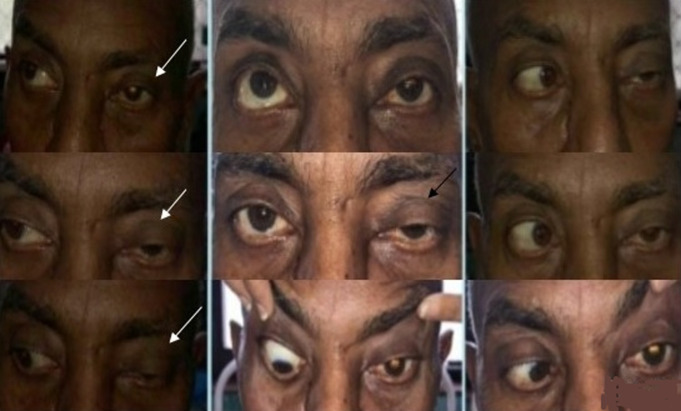
another patient presented with partial ophthalmoplegia (white arrow) and ptosis (black arrow)

**Figure 3:**
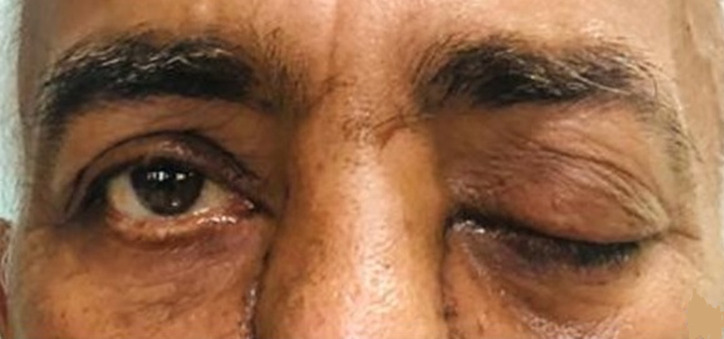
complete ptosis due to ROCM in the patient shown in figure 2

**Data sources/measurement:** the TRAMB was administered primarily as the only local ocular treatment in patients with reasonably preserved visual acuity with ophthalmoplegia, orbital apex syndrome and in subjects with total ophthalmoplegia. Medically unfit patients who were unable to undergo extensive debilitating surgeries under general anaesthesia and subjects who had preserved visual acuity but demonstrated limitation in ductions also received TRAMB. Patients showing clinical signs of orbital involvement in the form of frozen globe, chemosis, congestion, visual acuity amounting to perception of light/no perception of light to finger counting at 6 metres atleast {if snellen chart assessment of visual acuity not feasible- in debilitated and non-ambulatory patients}) and those with radiological features suggestive of invasive fungal sinusitis (Figure 1, Figure 2, Figure 3) were administered retrobulbar injection of emulsion type/lipid complex type of AMB (0.6 ml of 5mg/ml of emulsion type/lipid complex Amphotericin B with 0.4 ml of distilled water to a maximum of 1ml of undiluted Amphotericin B {5 mg/ml}). The average number of RAMB injections was 4.42 ({1-14 injections}, Table 3). One injection was given to one patient in stage 4 who underwent orbital exenteration subsequently. Two injections were given in 4 patients in stage 4 of which one patient was subjected to orbital exenteration. Rest all patients received three or more injections of RAMB (Table 3). Average duration between two sessions of 3-5 injections in each session was 9.42 days (3 to 16 days) in 28 patients (3-5 days in 5 patients; 6 to 10 days in 10 patients; and > 10 days in 13 patients). The maximum no. of RAMB injections were administered to patients in stage 3c followed by stage 3b, 4 and 3a. Intravenous administration of AMB (lipid complex type/emulsion type/liposomal type} based on its availability, kidney function status of the patient and intravenous antibiotics continued. Patients who were allergic to AMB were subjected to a test dose prior to the retrobulbar injection. Injection in the concentration of 0.5-0.5 mg/ml was used. In majority of the patients, two cycles with 3-5 injections in each cycle were given in the affected eye along the inferior orbital margin in the intraconal retrobulbar space. Local anaesthesia with lignocaine was infiltrated in all eyes prior to retrobulbar injection. Consecutive doses were then distributed over time depending upon the ocular response post retrobulbar injection. In some of our studied patients who demonstrated increased periocular oedema and conjunctival chemosis, retrobulbar injections were repeated once the inflammation subsided. A gap of 3-5 days between repeat RAMB injections was given to minimize the local inflammatory side effects of AMB.

**Table 3:** stage wise retrobulbar amphotericin B injections administered and outcome

Staging	No. of retrobulbar injections received	Average No. Of retrobulbar	Total systemic AMB dose (avg)	Exenteration	Stable/ exenteration/ sick/death
3A (n=10)	01 injections—1 02-03 injections--29 04-05 injections--15 06–10 injections--14 >10 injections --2	3.6	5035	0	10/0/1/0
3B (n=9)		4.77	2944.44	0	9/0/0/0
3C (n=21)		5.28	6733.33	11	10/11/0/2
4 (n=21)		3.80	5600	5	11/5/3/1

Patients who had undergone sinonasal debridement but showed progressively increasing orbital disease (marked proptosis, frozen globe, vision with perception of light or worse) with extensive radiological involvement despite receiving RAMB as well as intravenous AMB or posaconazole therapy (in the ones allergic to Amphotericin B) underwent orbital exenteration. Simultaneous sinonasal debridement with orbital exenteration was performed in patients who had extensive orbital involvement at presentation and were fit for general anaesthesia. These patients received 1-3 injections of RAMB until the day of surgery. The patients were followed up to ascertain the outcome of orbital exenteration in the form of systemic stabilization or an event in the form of death for a minimum period of three months. Detailed systemic evaluation of the patients was done and treatment for the same was given if needed. The tests that were conducted included: complete hemogram, liver function test (LFT), kidney function test (KFT), serum electrolytes, serum calcium, serum magnesium, glycosylated hemoglobin (HbA1c), lipid profile, electrocardiogram (ECG), chest X-ray. Contrast-enhanced computed tomography (CECT)/ non-contrast computerized tomography (NCCT)/ contrast-enhanced magnetic resonance imaging (CEMRI) were done for the patients depending on their systemic condition and type of imaging needed to evaluate the progression of the disease.

**Quantitative variables:** the quantitative variables included in the study comprised of total number of patients, grade of ocular motility, visual acuity in either eye, average age in years, number of retrobulbar injections administered and average AMB dose given systemically in milligrams. These were analysed statistically apart from the qualitative variables.

**Ethical issues:** there were no ethical issues related to the study. The study was approved by the Hospital Ethics Committee of Guru Teg Bahadur Hospital (GTBHEC), Delhi and adhered to the guidelines of the Declaration of Helsinki.

**Bias:** the study was retrospective in nature. Although the coding rules and definitions as an online appendix were provided to the investigator, some element of bias may exist while data is being coded from the case records. Patient sample not being representative of the entire population and the absence of needed variables from the records may add to bias.

**Statistical methods:** the data thus collected using the study tools was converted into a computer based spread sheet and analysed. The statistical analysis comprised of calculating means, standard deviation and proportions. The data was analyzed using appropriate software, Statistical Package for the Social Sciences (SPSS).

## Results

**Participants:** there were 61 patients in the study.

**Descriptive data:** 49/61 (68.85%) patients were known diabetics and 32/61 (52.45%) patients gave a positive history of recent COVID-19 infection. The majority of the risk groups in this study were having diabetes, immune-suppression due to recent COVID-19 infection and injudicious administration of steroids. All patients tested positive for fungal hyphae on KOH from the specimens retrieved from diagnostic nasal endoscopy. Due to non-availability of MRI machine at our center, patients were subjected to CECT orbit, paranasal sinus and brain and disease grading done accordingly. None of the patients presented with isolated involvement of the nasal mucosa. All of the study subjects suffering from ROCM presented with stage 2 or higher. Out of 61 patients, stage 3a, 3b, 3c, 4 was seen in 10, 9, 21, 21 patients respectively. Presenting visual acuity of no light perception was noted in 33 eyes of 61 patients. Fifty-five eyes of 61 patients showed visual acuity ranging from less than 6/60 to 6/36. Six eyes of 61 patients had a visual acuity of 6/24 to 6/9.

**Main results:** in this study, patients with visual acuity ranging from perception of light to below 6/60 were 26 in number, of which one patient gained one line of visual acuity and improved to 6/36 after TRAMB administration. The patients with a visual acuity of 6/60 or better remained stable during the entire follow-up. None of the patients deteriorated to a visual acuity of no light perception when compared to their presenting visual acuity.

**Figure 4:**
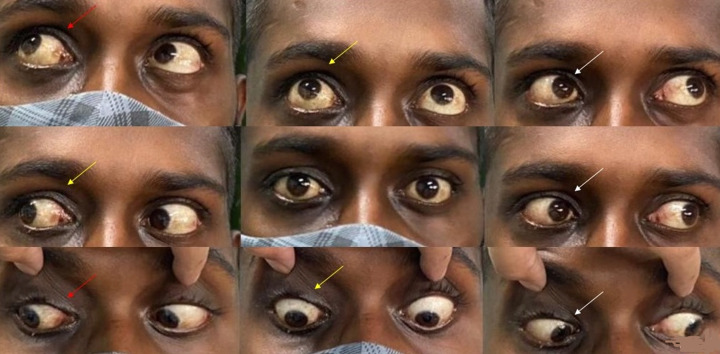
following TRAMB in patient described in figure 1, complete recovery of ocular signs was noted. Dextroelevation, dextroversion and dextrodepression improved in the right eye (red arrow), chemosis, proptosis, elevation and depression in the right eye (yellow arrow) improved and so did all the movements in left gaze in the right eye (white arrow)

**Figure 5:**
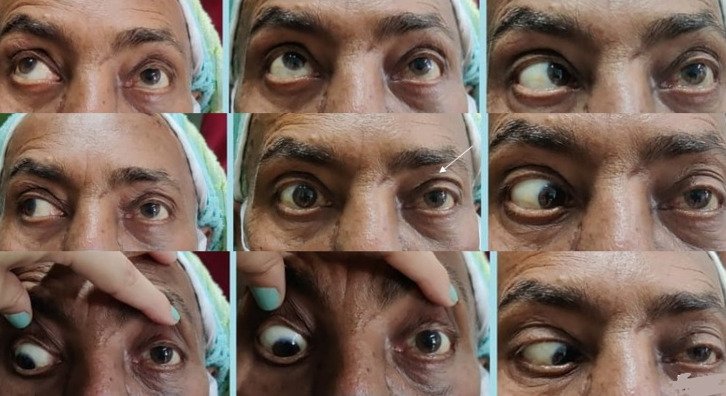
recovery in ocular signs was also noted in patient described in figures 2, 3 (white arrow, showing improvement in ptosis)

**Figure 6:**
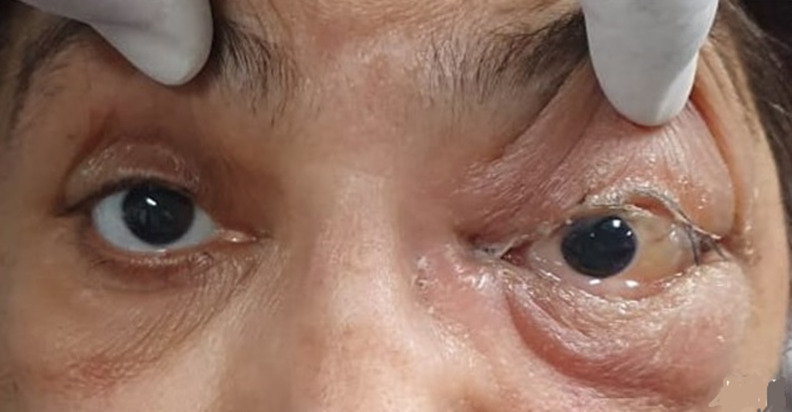
a patient developed conjunctival chemosis and periocular swelling following TRAMB

**Figure 7:**
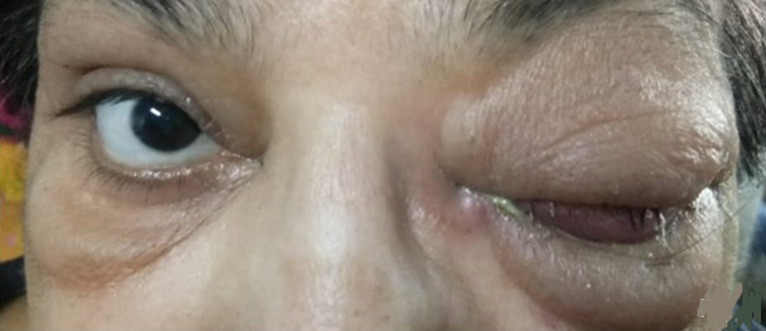
a patient developed conjunctival chemosis and periocular swelling following TRAMB

Proptosis improved in 11 patients (Figure 4), ptosis improved in nine patients (Figure 5), complete ptosis, proptosis and gaze improvement seen in one patient and 11 patients showed gaze improvement. Two out of 11 patients showed complete improvement of abduction and elevation. Twelve patients remained stable and did not progress further. Sixteen patients showed minimal improvement in orbital disease and underwent exenteration due to progression of orbital disease and systemic toxicity. Seventeen patients underwent external sinus debridement only. Two patients did not undergo sinus debridement due to systemic co-morbidities and were unfit for anesthesia. All studied patients underwent endoscopic/external sinus debridement. Endonasal orbital decompression along-with sinus debridement was performed in fifteen patients. The ones who underwent endoscopic debridement had to undergo external debridement as a second surgery due to progression of disease. Two surgeries for sinus debridement were performed in three patients. Seven patients underwent craniotomy for frontal sinus debridement along with orbital decompression. Forty patients received intraoperative TRAMB during external sinus debridement. On an average, 4.42 injections of TRAMB were given to almost all of our patients. Mortality was 4.91 percent (three patients). Two out of three patients succumbed to illness as they were not fit for administration of anaesthesia in view of systemic condition. One patient who underwent external sinus debridement twice along-with TRAMB died due to kidney failure. The complication rate of TRAMB injection was seen in seven patients (11.47%). One patient developed severe proptosis and conjunctival congestion due to progression of disease. Three cases developed increased periocular swelling (Figure 6, Figure 7) and another three of the seven who had complications following TRAMB developed conjunctival chemosis (Figure 7). None of these patients developed a reduction in their visual acuity. However, all these seven patients improved on further follow up.

**Outcome data:** Table 3 shows the distribution of outcome following TRAMB. Thirty-five (57.37%) patients showed complete recovery of the orbital and ocular inflammation. The disease was stable in eight patients, 16 (26.22%) patients underwent exenteration and three succumbed to illness. In stage 3a, all patients showed improvement. In stage 3b, 8 showed improvement and one remain stable. In stage 3c, 10 (47.61%) patients improved, 11 underwent orbital exenteration and 2 died. In stage 4, 3 patients showed improvement, 11 (52.38%) remained stable, 5 patients got exenterated and one succumbed to the illness.

## Discussion

The average age of the patients in this study was 51.4 years (25 to 77 years) with a male preponderance which was comparable to the age of the subjects in a multicentric Indian study and also in the study conducted by Muthu *et al*. [2]. A relatively young age at presentation with a surge in the number of cases of ROCM amidst COVID-19 pandemic points to the immune alteration caused by infection with SARS-CoV-2 along-with diabetogenic effect of the novel coronavirus. Transcutaneous administration of amphotericin B (TRAMB) is a minimally invasive procedure that can be used alternatively to surgical treatment with favorable outcome [33, 35-39].

Many reports exist in literature but the sample size is not robust enough and as large (61 patients, 122 eyes) as in our study to emphasize the efficacy and usefulness of TRAMB in orbital involvement in ROCM as a globe saving measure. Seiff *et al*. in their series of 7 cases of invasive fungal rhino-orbital infections showed the efficacy of local AMB irrigations when performed using a fenestrated, 10-Fr rubber catheter along-with sinus debridement as an effective means to control sino orbital fungal infections [40]. The fungal progression stopped or regressed in 6 patients and only 1 patient required exenteration. Research work by Evan Kalin Hajdu *et al*. [27] further highlighted the role of local AMB irrigation and TRAMB apart from the role of orbital exenteration, conservative debridement with local irrigation, and TRAMB. Guidelines proposed by a team of Indian Ophthalmologists related to post COVID-19 mucormycosis suggest that TRAMB has a definitive role in early orbital involvement in post COVID-19 mucormycosis [1, 2].

Retrobulbar administration of amphotericin B and its adjunctive use is not extensively reviewed in treating sino-orbital infection [35]. Four out of five patients received TRAMB as a globe saving measure in a multicentric Indian study that included 127 post COVID-19 mucormycosis cases [31]. This report described the use of deoxycholate variety of AMB injection. Sinonasal debridement in conjunction with intraoperative irrigation of amphotericin B has demonstrated fairly good outcomes in a small series of cases published in literature [27, 41-44]. A patient of ROCM and diabetic ketoacidosis received intraconal injection of amphotericin B (1 mg/ml) twice daily for 9 days along-with intravenous amphotericin and exenteration was avoided [44]. Local injection of amphotericin B has worked well in treating fungal infections of the joints, lungs and nasal cavity [45-47]. Cotton soaked pledgets of amphotericin B have benefitted patients undergoing functional endoscopic sinus surgery based on the study by Sarkar *et al*. apart from the routine systemic administration of amphotericin B and sinus debridement [30]. Local irrigation of orbital tissues with amphotericin B via a catheter is described [43, 47].

Amphotericin B gets accumulated maximally at the site of application. Two cases of rhino-orbital mucormycosis treated with local irrigation and systemic treatment with AMB was reported by Fleckner and Goldstein in 1969 [48]. Four times daily orbital irrigation with AMB at a concentration ranging from 0.25 to 1.25mg/ml (1 to 15ml) over a period of 5 days to 4 weeks is described in literature [40, 41, 44, 49-51]. Baginski *et al*. showed that a modest volume of 5ml of AMB (1mg/ml) infused into the orbit daily results in successful outcome [52]. Placement of a wide bore intravenous cannula near the orbital apex can help in more diffuse delivery of the drug and reduces the need for repetitive injections. Recently, Muthu *et al*. subjected 82 eyes of 75 patients to daily injections of AMB using an 18-G cannula with an injection port inserted into the orbital space and held by sutures where the drug was delivered for 5 days [2]. They used liposomal variety of AMB in their study. A satisfactory response in terms of regression of the orbital disease and a good patient compliance was reported. The authors concluded that TRAMB in conjunction with systemic antifungals and endoscopic sinus debridement worked as a potential globe-sparing intervention for orbital mucormycosis [2]. This modality can be considered for management of early or minimal orbital involvement in patients with mucormycosis [2].

Our result in 122 eyes (RE-61, LE-61) of 61 patients is the largest study in terms of sample size that describes a satisfactory clinical response to retrobulbar administration of deoxycholate/emulsion variety of AMB. The entire country witnessed the epidemic of COVID-19 associated ROCM amidst the pandemic with Northern India facing the biggest challenge as the number of patients presenting to the tertiary care centers was huge. Patients received deoxycholate/emulsion variety of intravenous AMB in the initial phase of the epidemic as the liposomal formulation was not available in significant quantity due to a surge in the number of cases requiring this drug. Intravenous/oral posaconazole therapy continued systemically along-with intravenous deoxycholate/emulsion type AMB. This combination drug therapy was considered as a promising strategy to minimize the toxicity of AMB by reducing the dose of deoxycholate variety dose and provide a synergistic effect [53]. Combined administration of posaconazole along-with amphotericin B or posaconazole monotherapy in AMB intolerant patients has shown lower risk of exenteration possibly due to pharmacological synergy between the two antifungal agents [54-56].

The systemic safety and clinical efficacy of liposomal AMB is well known. Once available in adequate quantity, the drug had to be utilized for systemic administration. In the meantime, RAMB (deoxycholate/emulsion type) continued and authors witnessed a satisfactory clinical response. The authors evolved in their management strategy and decision making when such large numbers of patients presented to the dedicated center for management of mucormycosis. Based on literature where continuous administration of local AMB resulted in satisfactory clinical response, a step ladder approach evolved wherein three injections of RAMB were given on three continuous days. The decision for RAMB was based on clinical signs of orbital involvement in the form of ptosis, proptosis, visual acuity, ophthalmoplegia apart from signs of orbital inflammation (pain and eyelid swelling). Radiological involvement (CECT/MRI) in the form of extraconal fat stranding, thickening and bulkiness of the extraocular muscles and uptake of contrast by the orbital tissues gave a pointer that the local administration of AMB in the retrobulbar intraconal space would be beneficial. Involvement of the orbital apex was ascertained clinically and radiologically and TRAMB injection was given in an attempt to contain the disease to the orbit and limit intracranial spread. Patients presenting with a stage 4 disease received RAMB injections to reduce the fungal load in the orbit while the patient awaited sinonasal debridement with or without a neurosurgical intervention. If the patient tolerated three injections well without showing any signs of localized orbital inflammation, a repeat set of three more injections was given. Over time, the number of such injections was increased to greater than three upto five in one sitting.

The deoxycholate form of AMB has been used for retrobulbar injections with good results as reported in literature. It reduces the risk of nephrotoxicity along-with a reduction in the cost of injections unlike when the liposomal form is used. Management of patients suffering from ROCM using TRAMB serves as a rescue therapy and helps in buying out time for systemic stabilization of the patient until considered fit for sinus debridement and or orbital exenteration. Treatment with TRAMB is an effective way to halt deterioration in patients and limit intracranial extension of the disease when the disease is limited to the orbit. Although RAMB is being used off-label till date, certain risks in the form of local tissue inflammation locally and neurotoxicity are known [6, 57-58].

In our cohort of patients, not many patients lost their presenting visual acuity. A few subjects had a gain in their visual acuity which could be the result of control in their orbital disease that could be the effect of preservation of optic disc perfusion following administration of RAMB. This was also the observation in the study reported in literature [57]. Orbital exenteration is reserved for patients with extensive orbital disease however direct survival benefit has not been demonstrated based on the results of the published meta-analysis and retrospective studies [29, 32-33]. This limited benefit of orbital exenteration in terms of overall survival makes it challenging for the attending ophthalmologist to perform this surgery. This difficult decision is made when the extent of the disease and the risk of mortality outweigh the desire to keep the patient´s globe in place. It was also observed that orbital exenteration rates reduced considerably following administration of RAMB injection. This fact is also reported in literature where 9.1% of the patients underwent orbital exenteration in a study by Ashraf *et al*. [57]. A modified step ladder pattern of administration of RAMB was followed in patients included in the research study by Ashraf *et al*.wherein a similar risk of mortality was noted in the individuals who received RAMB when compared with the controls however the risk of orbital exenteration was low. The authors proposed reasons for this difference. First was the delivery of antifungal medication directly to the infected orbital tissues as the systemic amphotericin B penetration into the infected tissues is low. This reflects with reduction in the need for mandatory debridement. Secondly, the orbital tissue is supplied with a rich yet highly redundant vasculature. Orbit might be more responsive to systemic therapy however for moderate orbital disease; the authors concluded that initial TRAMB rather than exenteration resulted in a lower mortality with the preservation of the orbit.

The authors could not use the liposomal variety of amphotericin B for retrobulbar administration in their large cohort of patients presenting to a dedicated tertiary care centre at one point of time. This was a limiting factor in the study where the effect of liposomal variety of retrobulbar amphotericin B could not be studied. The decision for administration of retrobulbar injection was based predominantly on contrast enhanced CT images as performing MRI in large number of patients was not feasible. The results of this study are chiefly based on CT findings. Although a limitation of this study, it is still encouraging to note that decision to administer RAMB can still be made on CT findings. This is one of the highlights of this study as also the use of deoxycholate/emulsion formulation of amphotericin B that gave satisfactory results.

## Conclusion

Retrobulbar injection of amphotericin B along-with systemic antifungal therapy with sinonasal debridement in patients of ROCM may help in stabilization of orbital disease as also it may reduce the number of patients requiring orbital exenteration.

### What is known about this topic

- TRAMB is considered as one of the treatment options for orbital mucormycosis;
- Most of the studies regarding TRAMB have used liposomal formulation of amphotericin B;
- Majority of the research articles published in literature have administered 3-5 injections of retrobulbar amphotericin B to the included subjects.

### What this study adds

- TRAMB helps in stabilization of the orbital disease apart from complete resolution of orbital signs in the form of proptosis, ptosis and ophthalmoplegia in few cases when administered along-with systemic amphotericin B;
- Retrobulbar administration of Deoxycholate/emulsion variety of amphotericin B may be considered as an alternative to liposomal amphotericin B formulation;
- CT scan may guide decision making for retrobulbar injection of amphotericin B. This is one of the few published original research articles involving a large number of eyes requiring TRAMB.
